# Rating of perceived exertion versus heart rate for isometric exercise prescription: Reliability and agreement study

**DOI:** 10.1590/1516-3180.2024.0092.R1.29072024

**Published:** 2025-03-17

**Authors:** Paulo Henrique de Melo, Anderson Cavalcante, Jessika Karla Tavares do Nascimento Faustino Silva, José Lucas Porto Aguiar, Jefferson Maxwell De Farias Silva, Theo Victor Alves Soares Rêgo, Raphael Mendes Ritti-Dias, Breno Quintella Farah

**Affiliations:** I Post-graduate Program in Rehabilitation Sciences, Universidade Nove de Julho (Uninove), São Paulo (SP), Brazil.; II Associated Post-graduate Program in Movement Sciences, Physical Education Department, Universidade Federal Rural de Pernambuco (UFRPE), Recife (PE), Brazil.; III Post-graduate Program in Rehabilitation Sciences, Universidade Nove de Julho (Uninove), São Paulo (SP), Brazil.; IV Post-graduate Program in Physical Education, Physical Education Department, Universidade Federal de Pernambuco (UFPE), Recife (PE), Brazil.; V Physical Education Department, Universidade Federal de Pernambuco (UFPE), Recife (PE), Brazil.; VI Physical Education Department, Universidade Federal de Pernambuco (UFPE), Recife (PE), Brazil.; VII Professor, Post-graduate Program in Rehabilitation Sciences, Universidade Nove de Julho (Uninove), São Paulo (SP), Brazil.; VIII Department of Physical Education, Associated Postgraduate Program in Movement Sciences, Universidade Federal Rural de Pernambuco (UFRPE), Recife (PE), Brazil; Professor, Postgraduate Program in Physical Education, Universidade Federal de Pernambuco (UFPE), Recife (PE), Brazil; Postdoctoral, Postgraduate Program in Rehabilitation Sciences, Universidade Nove de Julho (Uninove), São Paulo (SP), Brazil.

**Keywords:** Exercise, Resistance training, Observer variation, Reproducibility of results, Isometric exercise, Reproducibility, Squat exercises

## Abstract

**BACKGROUND:**

Previous studies have shown that isometric exercise training reduces systolic blood pressure by approximately 8 mmHg and diastolic blood pressure by approximately 4 mmHg in both normotensive and hypertensive individuals. However, the prescription of isometric exercises can be based on the rating of perceived exertion (RPE) or heart rate (HR) obtained during the maximal incremental isometry test. The reliability and agreement of this test have not been assessed.

**OBJECTIVES:**

To analyze the reliability and agreement indicators of HR and RPE during isometric wall squat incremental tests.

**DESIGN AND SETTING:**

A reliability and agreement study was conducted at Universidade Federal de Pernambuco.

**METHODS:**

Twenty-eight healthy subjects (54% men, 26 ± 5 years) performed two isometric wall squat incremental tests. The test began with a knee joint angle of 135° (knee and leg) progressively reduced by 10° at each stage. Each stage lasts 2 minutes or until voluntary exhaustion. The HR and RPE were obtained during the tests. Reliability and agreement were established using test-retest (paired t-test or Wilcoxon test), intraclass correlation coefficient (ICC), standard error of measurement (SEM), coefficient of variation (CV), and Bland-Altman plots.

**RESULTS:**

The HR and RPE increased significantly during both tests. The HR and RPE at each stage were similar between the two test sessions (P > 0.05). Both HR_max_ (ICC: 0.695, P = 0.002, SEM = 8.1 bpm and CV = 5.8%) and RPE_max_ (ICC: 0.525, P = 0.036, SEM = 0.4 and CV = 3.6%) presented similar reliability indicators, and no statistically significant differences were obtained between the two test sessions (P > 0.05). The Bland-Altman plots indicated good agreement between HR_max_ and RPE_max_.

**CONCLUSION:**

HR and RPE showed similar reliability and agreement during the isometric wall squat incremental test.

## INTRODUCTION

Isometric exercise training has resulted in clinically significant reductions of approximately 8 mmHg in systolic blood pressure and 4 mmHg in diastolic blood pressure in both normotensive and hypertensive individuals.^(1,3,4)^ These reductions are equal to or greater than those observed in other forms of exercise, such as aerobic or dynamic resistance training.^2^

In contrast to other forms of isometric exercises, such as handgrip and leg extension, wall squat training does not require equipment (e.g., isokinetic and handgrip dynamometers),^5,6,^ which makes it attractive for performance in different settings. Isometric wall squat training has been prescribed as a percentage of the heart rate peak (95% HR_peak_) achieved during an incremental isometric exercise test.^2,4,7^ Recently, Lea et al.^8^ also demonstrated that prescribing isometric wall squat training based on the rating of perceived exertion (RPE) had a comparable effect on blood pressure as when prescribed based on 95% HR_peak_. Both methodologies demonstrated a low coefficient of variation (CV) during an isometric wall squat incremental test,^9,10,11,12^ suggesting good agreement between the measurements.

Despite these favorable agreement indicators, robust metrics, including standard error of measurement (SEM) and the limits of agreement of Bland-Altman plots^13, 14^ remain unknown. Furthermore, no study has demonstrated the intraclass correlation coefficient (ICC) for HR and RPE during the test. Therefore, despite being widely used^15^, it is still unknown whether the isometric wall squat incremental test is reliable.

## OBJECTIVE

This study aimed to analyze the indicators of reliability and agreement of HR and RPE during the isometric wall squat incremental test.

## METHODS

### Participants

Healthy adults of both sexes, including those who had not been performing recreational resistance training, were recruited via social media and flyers distributed near the university. Participants were eligible if they met the following criteria: a) no history of cardiovascular disease or diabetes, b) absence of osteoarticular injuries or conditions that would hinder the performance of squat exercises, c) nonsmokers, and d) not taking medications or supplements that could alter hemodynamic variables. Participants who did not complete both tests were excluded.

All the participants provided written informed consent to participate in the study, which the Institutional Review Board approved (#3.558.606) on September 6, 2019, per the Brazilian National Research Ethics System Guidelines.

### Protocol

All participants who consented to participate in the study attended two laboratory visits. Demographic information, medical history, and medication use details were collected during the initial visit. Additionally, height and weight were measured following standardized protocols, and body mass index was calculated. Subsequently, the participants were familiarized with the testing procedures and underwent an isometric wall squat incremental test. On the second assessment day, after at least 48 hours, participants repeated the isometric wall squat incremental test at the same time as on the first day.

For the incremental isometric wall squat test, the participants remained upright with their backs against a wall, feet parallel and shoulder-width apart, and hands by their sides. A clinical goniometer (Trident, Brazil) with a protractor divided into degrees was attached to each participant’s knee using an elastic Velcro strap to control the angle of movement.

**Figure 1:**
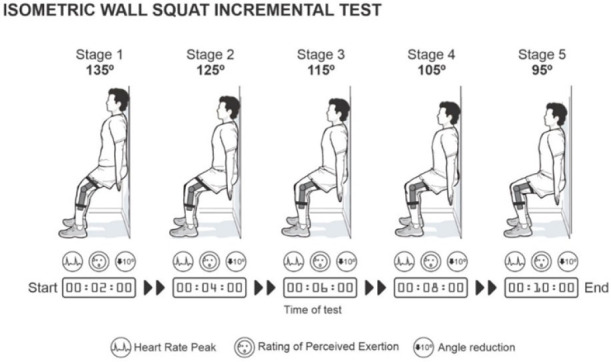
Knee joint angles used for the five consecutive 2-min stages of the isometric wall squat incremental test.

The test has five progressive stages.^7,9^ The first stage began at 135° of knee flexion, and the participants were instructed to hold this position for 2 min. Once each stage was completed, the knee joint angle decreased by 10°. Stage progression passes without intervals through the following angles: 125º, 115º, 105º, and 95º (Figure 1). The test was considered complete when the participant concluded the 5^th^ stage or could not maintain the knee joint angle for 2 min. During the test, the evaluator provided verbal encouragement and instructions on maintaining normal breathing and avoiding the Valsalva maneuver.

HR = heart rate; RPE = rating of perceived exertion

During the test, HR was continuously measured using a cardio monitor (Polar Vantage M2, Polar Electro Oy, Finland). HR data were extracted from the cardiac monitor and synchronized using Polar Flow syn*c software*. The average HR of the final 30 s in each stage^12^ was considered and the highest HR value was considered HR_max_.

RPE was recorded using the Isometric Exercise Scale.^10,11^ Standardized scaling and anchoring instructions were given to each participant before the test, as previously described.^10,11^ Participants were asked to report the RPE in their active muscles in the final 10 seconds of each stage of the isometric wall squat incremental test. Participants were cued to give their ratings using the standardized question, “How hard do you feel your muscles are working?” The scale was positioned in the full view of the participants for the entire test. The RPE_max_ was defined as the highest value obtained during the test.

### Statistical analysis

Data were stored in Microsoft Excel (Microsoft, Redmond, Washington, United States, 2016) and analyzed using SPSS for Windows version 25 (IBM Inc., Chicago, Illinois, USA). The normality of the data distribution was analyzed using the Shapiro-Wilk test. Parametric data are presented as mean ± standard deviation (SD), and non-parametric data are presented as medians (interquartile ranges).

The paired t-test and Wilcoxon test were used to compare the HR and RPE responses on the two days of the isometric wall squat incremental test. The reliability of the isometric wall squat incremental test was analyzed by ICC (3, K) based on the average rating, absolute agreement, and two-way mixed effects model as suggested^16,17,18^. Agreement measures were calculated using CV, Bland-Altman plot, and SEM. A P value of < 0.05 was used to establish statistical signiﬁcance.

## RESULTS

Thirty participants were enrolled in this study. Two participants completed only one day of testing and were excluded from the study. Thus, 28 healthy individuals (15 male and 13 female) were included in the analysis. Nine participants performed resistance exercises recreationally. Participants’ characteristics are presented in Table 1.

**Table 1:** Sociodemographic characteristics of the participants (n = 28)

	Values
Male, %	53.6
Physically active, yes %	32.1
Age, years	26.0 ± 4.8
Height, cm	67.0 ± 13.7
Weight, kg	1.70 ± 0.1
BMI, kg/m²	23.2 ± 3.5

Values are presented as frequency or mean ± standard deviation

Fifteen participants completed the test in the same stages on both days, and three participants finished the test in the final stage (95º angle) on both days. The reliability values of HR_max_ and RPE_max_ during the isometric wall squat incremental tests are listed in Table 2. No significant differences were observed between Tests 1 and 2 for HR_max_ and RPE_max_.

**Table 2:** Intraclass correlation coefficient, standard error of measurement, coefficient of variation, and minimum detected difference of heart rate and rating of perceived exertion in the isometric wall squat incremental test

	Test 1	Test 2	P	ICC	P	SEM	CV	Bias ± SD	95%LoA
HR_max_	139 (17)	137 (24)	0.867	0.695	0.002	8.1	5.8	-0.39 ± 14.6	-29.0 to 28.3
RPE_max_	9.5 (1.0)	10.0 (1.0)	0.212	0.525	0.036	0.4	3.6	-0.18 ± 0.67	-1.49 to 1.13

Data are presented as median (interquartile range); SD = standard-deviation; HR_max_ = the highest heart rate value during the isometric wall squat incremental test; RPE_max_ = the highest rating of perceived exertion value during the isometric wall squat incremental test; ICC = intraclass correlation coefficient; SEM = standard error of measurement, CV = coefficient of variation; 95%LoA = 95% limit of agreement.

**Figure 2:**
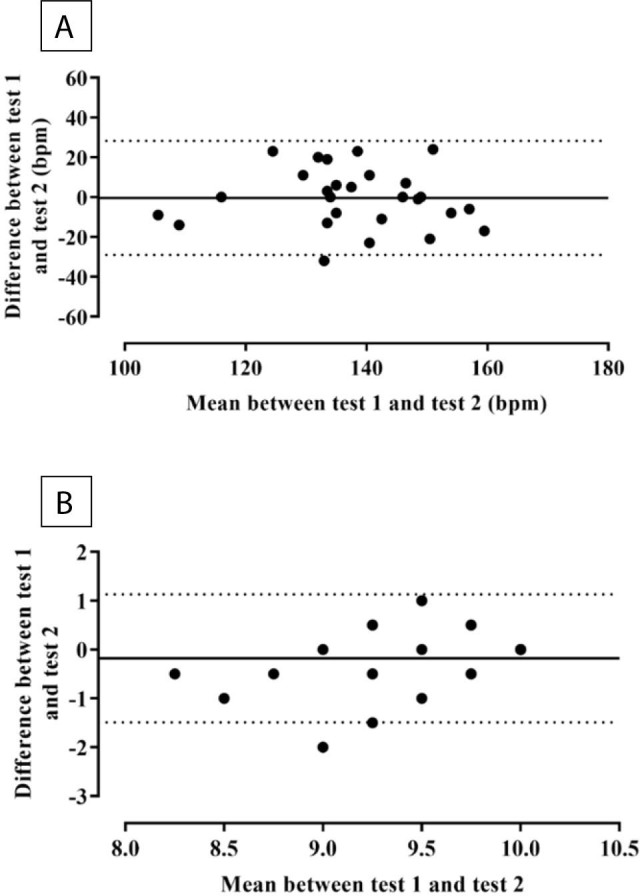
Bland – Altman plots for HR_max_ (Panel A) and RPE_max_ (Panel B) between Test 1 and Test 2 during the isometric wall squat incremental test.

Figure 2 illustrates the agreement between Tests 1 and 2 for HR_max_ and RPE_max_. The Bland–Altman plots indicated good agreement for both HR_max_ and RPE_max_.

HR_max_ = The highest heart rate during the isometric wall squat incremental test;

RPE_max_ = the highest rating of perceived exertion value during the isometric wall squat incremental test.

The reliability and agreement indicators of HR and RPE during the isometric wall squat incremental tests are shown in Table 3. No significant differences (P > 0.05) were observed between tests 1 and 2 in the isometric wall squat incremental test.

**Table 3:** Intraclass correlation coefficient, standard error of measurement, coefficient of variation and minimum detected difference of heart rate and rating of perceived exertion in each stage of the isometric wall squat incremental test

Stages	n	Test1	Test2	P	ICC	P	SEM	CV	Bias ± SD	Number of outliers	95%LoA
**Heart rate**											
1^st^	28	102 ± 14	107 ± 11	0.067	0.659	0.002	7.2	7.2	-4.46 ± 12.39	1	-28.74 to 19.81
2^nd^	28	115 ± 13	117 ± 11	0.496	0.594	0.012	8.4	6.8	-1.71 ± 13.15	1	-27.49 to 24.06
3^rd^	27	131 ± 15	131 ± 12	0.988	0.766	< 0.001	6.0	5.4	0.04 ± 12.36	1	-24.19 to 24.27
4^th^	12	134 ± 13	134 ± 16	0.638	0.682	0.041	5.8	5.3	-2.00 ± 14.30	1	-30.03 to 26.03
**Rating of perceived exertion**											
1^st^	28	2.0 (2.0)	1.3 (1.0)	0.135	0.879	< 0.001	0.3	41.1	0.28 ± 0.92	1	-1.53 to 2.08
2^nd^	28	5.3 (3.4)	4.5 (3.5)	0.750	0.754	< 0.001	1.0	25.8	-0.16 ± 2.03	1	-4.14 to 3.82
3^rd^	27	8.5 (3.0)	8.5 (2.5)	0.914	0.831	< 0.001	0.5	9.3	0.04 ± 1.3 1	1	-2.54 to 2.62
4^th^	12	9.3 (1.4)	9.8 (1.4)	0.729	0.922	< 0.001	0.2	3.5	-0.08 ± 0.60	0	-1.25 to 1.09

Data presented as mean ± standard deviation or median (interquartile range); HR = The average the HR of the last 30 seconds in each stage; RPE = Rating of perceived exertion in the previous 10 seconds of each stage; ICC = intraclass correlation coefficient; 95%CI = confidence interval 95%; SEM = standard error of measurement, CV = coefficient of variation, 95%LoA = 95% Limits of Agreements.

## DISCUSSION

The results of the current study demonstrated that both the HR and RPE presented comparable reliability and agreement in monitoring the intensity of isometric wall squat exercises in healthy adults. We did not observe any statistically significant differences between the two days of the incremental wall squat test for either HR_max_ or RPE_max_. In addition, no differences were found for either RPE or HR at any stage, consistent with findings from previous studies,^(10,11,12)^ suggesting no systematic bias.^14^

The ICC has been considered the primary reliability measure for continuous outcomes.^13,14^ In the current study, the ICC values were 0.695 for HR_max_ and 0.525 for RPE_max_. Although previous studies^10,11^ have assessed the ICC for HR and RPE during exercise sessions at various angles, none have evaluated the isometric wall squat test, the main parameter used for wall squat exercise prescription. In addition, the ICC values during each test stage showed a high proportion of actual variance, suggesting that the data presented good reliability for HR and that the variability was due to individual differences. This indicated that the measurements were relatively reliable and replicable^14^.

The CV of HR_max_ values obtained in this study (5.8%) were in line with previous studies that report results ranging from 3.6%^9^ to 6.3%.^12^ Conversely, none of these studies assessed the CV for RPE during the isometric wall squat test. The only data referred to the RPE during the different stages of the exercise sessions, ranging from 54% (angle of 135º) to 4.5% (angle of 95º). This is consistent with our findings of 41.1% (angle of 135º) to 3.5% (angle of 105º), indicating lower CVs at lower angles.

SEM has also been inadequately explored during isometric wall squat tests. Only Lea et al.^10^ presented SEM data for HR and RPE during an exercise session and observed values of 2.4 bpm and 0.65, respectively. We observed an HR_max_ of 8.1 bpm and an RPE_max_ of 0.4. These results suggest that the SEM for both the HR and RPE across tests and sessions is acceptable.

The current study is the first to describe the Bland-Altman limits of agreement during the isometric wall squat test. The results revealed consistent and adequate agreement between measurements, demonstrating differences characterized by low variability and the absence of systematic patterns for both HR and RPE. One outlier was observed in both methods, indicating that, in a few cases, wide variability in HR and RPE can occur between tests.

The present study has some limitations that should be considered when interpreting the results. Some participants did not complete the final stages of the incremental test, limiting their responses or data analysis. Furthermore, the participants self-reported regular physical activity, which could have led to a memory bias. Finally, the training histories of those undergoing recreational resistance training (32% of the participants) were not assessed. It is worth noting that the incremental test results may not fully represent a complete exercise session, and our study sample comprised exclusively of healthy young adults. Therefore, caution should be exercised when generalizing these findings to other clinical populations. However, the present study adds significant information to the scientific community regarding the reliability and agreement of HR and RPE during the isometric wall squat incremental test, which is an intensity-determining method.

## CONCLUSION

The reliability and agreement of the HR and RPE were similar, indicating that both methods presented similar psychometric properties for monitoring intensity in isometric wall squat training.
